# Human, viral or mutant human IL-10 expressed after local adenovirus-mediated gene transfer are equally effective in ameliorating disease pathology in a rabbit knee model of antigen-induced arthritis

**DOI:** 10.1186/ar1960

**Published:** 2006-05-16

**Authors:** Annahita Keravala, Eric R Lechman, Joan Nash, Zhibao Mi, Paul D Robbins

**Affiliations:** 1 Department of Molecular Genetics and Biochemistry, University of Pittsburgh, School of Medicine, Pittsburgh, PA 15261, USA

## Abstract

IL-10 is a Th2 cytokine important for inhibiting cell-mediated immunity while promoting humoral responses. Human IL-10 (hIL-10) has anti-inflammatory, immunosuppressive as well as immunostimulatory characteristics, whereas viral IL-10 (vIL-10), a homologue of hIL-10 encoded by Epstein Barr virus (EBV), lacks several immunostimulatory functions. The immunostimulatory characteristic of hIL-10 has been attributed to a single amino acid, isoleucine at position 87, which in vIL-10 is alanine. A mutant hIL-10 in which isoleucine has been substituted (mut.hIL-10) is biologically active with only immunosuppressive, but not immunostimulatory, functions, making it a potentially superior therapeutic for inflammatory diseases. To compare the efficacy of mut.hIL-10 with hIL-10 and vIL-10 in blocking the progression of rheumatoid arthritis, we used replication defective adenoviral vectors to deliver intra-articularly the gene encoding hIL-10, vIL-10 or mut.hIL-10 to antigen-induced arthritic (AIA) knee joints in rabbits. Intra-articular expression of hIL-10, vIL-10, and mut.hIL-10 resulted in significant improvement of the pathology in the treated joints to similar levels. These observed changes included a significant reduction in intra-articular leukocytosis and the degree of synovitis, as well as normalization of cartilage matrix metabolism. Our results suggest that hIL-10, vIL-10, and mut.hIL-10 are all equally therapeutic in the rabbit AIA model for treating disease pathology.

## Introduction

Rheumatoid arthritis (RA) is a debilitating, autoimmune disorder characterized by chronic erosive inflammation of the joints with invasive proliferation of synovial cells into the articular cartilage and attendant bone destruction. Pro-inflammatory cytokines, particularly tumor necrosis factor (TNF)-α, and IL-1 are thought to be important mediators that drive the pathophysiology of RA [1-4]. Considerable progress has been reported with the use of biological agents that mediate the pathogenesis of RA, including interleukin-1 receptor antagonist (IL-1Ra), antibodies to IL-1 and TNF-α, and soluble TNF-α receptors [5-10]. In particular, sTNF-R (Enbrel), anti-TNF antibody (Remicade), and IL-1Ra (Kinaret) are commercially available. Unfortunately, these protein-based biological agents have a short half-life, requiring weekly subcutaneous or intravenous delivery. Conversely, intra-articular transfer of the genes encoding immunomodulatory agents is an effective approach to achieve high, localized and sustained levels following a single treatment. Several studies in different animal models of RA clearly show that *in vivo *gene delivery to one diseased joint is highly effective in ameliorating disease not only in that joint, but in the contralateral joints as well [11-13].

One cytokine that has been of interest as a therapeutic for RA is IL-10. It is a key cytokine found in the human immune response that inhibits cell-mediated immunity and inflammation while promoting humoral responses [14]. It is a 35 kDa non-covalent homodimer produced by macrophages, B-lymphocytes and Th2 cells, and is a potent inhibitor of Th1 cytokines [15]. This activity accounts for its initial designation as a cytokine synthesis inhibition factor (CSIF) [16].

The actions of IL-10 are diverse in that IL-10 can be anti-inflammatory, immunosuppressive or immunostimulatory, depending upon the target cell. However, the principal function of IL-10 appears to be anti-inflammatory, limiting and eventually terminating inflammatory responses by inhibiting synthesis of monocyte and macrophage derived pro-inflammatory cytokines [15-19]. IL-10 also has the ability to inhibit the antigen-presenting function of monocytes/macrophages and dendritic cells through the down-regulation of MHC class II molecules and the co-stimulatory molecules B7 and intercellular adhesion molecule-1 (ICAM-1), classifying it as an immunosuppressive cytokine [20-24]. In addition to these activities, IL-10 has some immunostimulatory properties; it regulates growth and/or differentiation of B cells, natural killer cells, cytotoxic and helper T cells, mast cells, granulocytes, dendritic cells, keratinocytes, and endothelial cells. IL-10 plays a key role in differentiation and function of the regulatory T cell [25,26].

Human IL-10 (hIL-10) exhibits 73% homology with murine IL-10 (mIL-10) and 84% with the open reading frame of the Epstein-Barr virus (EBV), initially known as BCRF1 or now termed as viral IL-10 (vIL-10) [27]. vIL-10 shares many of the anti-inflammatory properties of mIL-10 and hIL-10, but lacks their immunostimulatory properties [24,25]. Isoleucine at position 87 of murine and human IL-10 is crucial for the immunostimulatory function. This amino acid in viral IL-10 is alanine. Studies have shown that by substituting isoleucine with alanine at position 87 in hIL-10, the immunostimulatory response could be abrogated, leaving the mutant human IL-10 (mut.hIL-10) biologically active with only immunosuppressive activity and receptor species specificity [28]. Various studies of inflammatory situations, in mouse tumor models, cardiac allograft experiments, and endotoxemic models, have all suggested a potential superiority of vIL-10 over hIL-10 as an immunosuppressive agent [29-31]. vIL-10 has also been tested in the antigen induced arthritis (AIA) rabbit model and the collagen induced arthritis mouse model and shown to confer a significant therapeutic effect [11,12,32,33].

In this study, we used the rabbit AIA model to compare directly the therapeutic efficacy of hIL-10, vIL-10, and mut.hIL-10, encoded by genes delivered intra-articularly *in vivo *by adenoviral vector. We demonstrate that expression of hIL-10, vIL-10 or mut.hIL-10 in the rabbit joints were similarly effective not only in preventing the progression of the disease by blocking leukocytic infiltration, but also reducing the degree of synovitis, as well as normalizing cartilage metabolism. These results suggest that the three variants of IL-10, hIL-10, vIL-10, and mut.hIL-10, are all equally therapeutic when delivered locally in the rabbit experimental arthritis model.

## Materials and methods

### Adenovirus vectors

The recombinant vectors used in this study were E1/E3 deleted replication-defective type 5 adenoviruses [34]. The cDNA encoding either hIL-10, vIL-10, mut.hIL-10 or enhanced green fluorescent protein (eGFP) was inserted into the E1 region with gene expression driven by the early promoter of the human cytomegalovirus. High titer recombinant adenoviruses (Ad.hIL-10, Ad.vIL-10, Ad.mut.hIL-10, and Ad.eGFP) were generated as described previously [35] by Cre-Lox driven homologous recombination and permissive replication in CRE8 cells, a 293 cell-line (ATCC, MD) that expresses Cre recombinase. Viral titers were determined by optical density at 260 nm (OD_260_) where 1 OD unit = 10^12 ^viral particles [36].

### Rabbits

Female New Zealand White rabbits, weighing approximately 5 to 6 lbs each, were purchased from Myrtles Rabbitry (Thompson Station, TN, USA) and housed in the Central Animal Facility at the University of Pittsburgh. The animals were acclimatized for three days before experimentation and were fed chow *ad libitum *and water. All animal experiments were conducted in accordance with NIH standards of animal care and the animal protocol used for this study was approved by the animal ethics committee of the University of Pittsburgh.

### Experimental protocol

The rabbits were sensitized by a series of two intradermal injections of 5 mg of chick ovalbumin (OVA; Sigma, St. Louis, MO, USA), emulsified in Freund's complete adjuvant (Pierce, Rockford, IL, USA) and Freund's incomplete adjuvant (Pierce) respectively, given 10 days apart [37]. Acute monoarticular arthritis was induced in both knee joints two weeks after the booster shot by intra-articular injection of OVA dissolved in 0.5 ml saline.

Twenty-four hours post-initiation of AIA, 5 × 10^9 ^particles of replication-defective adenovirus encoding either hIL-10, vIL-10, mut.hIL-10 or eGFP (control) was suspended in 0.2 ml sterile saline and injected into the joint space via the patellar tendon.

On days 3 and 7 post adenoviral delivery, the rabbit knee joints were lavaged by the injection of 1 ml of Gey's balanced salt solution (Gibco-BRL, Grand Island, NY, USA) into the joint space via the patellar tendon. After manipulation of the joint, the needle was reinserted and the fluid aspirated. Leukocytes suspended in the recovered lavage fluid were counted using a hemocytometer. Levels of IL-10 in recovered lavage fluids and sera were measured using a cytokine ELISA kit (Pierce Endogen, Rockford, IL, USA).

### Cartilage metabolism

To quantify the glycosaminoglycans (GAGs) released into the joint space as a result of cartilage proteoglycan breakdown, the lavage fluid from days 3 and 7 were first centrifuged at 14,000 × g for 10 minutes to get rid of all the debris, and the supernatant recovered. Aliquots (100 μl) of this supernatant were treated with papain to enzymatically cleave the proteins. Then, 20 μl of papain suspension (Type III, 19 U/mg protein; Sigma) was added to 1 ml of buffer containing 10 mM sodium EDTA and 0.4 M sodium acetate, pH 5.2. This papain solution (100 μl) was added to 100 μl of lavage fluid supernatant and incubated overnight at 60°C. Papain was then inactivated by iodoacetic acid (Sigma) to a final concentration of 4 mM. The samples were centrifuged at 14,000 × g for 10 minutes, and the supernatant transferred to fresh tubes; 2 U of hyaluronate lysase (Sigma) was added and the samples incubated at 37°C overnight. Sulfated GAG concentrations were measured as previously described [38] by a colorimetric dye binding assay using 1,9-dimethylmethylene blue reagent.

To measure the rate of proteoglycan synthesis, the harvested knees were dissected, and fragments of articular cartilage were shaved from the femoral condyles. Cartilage fragments weighing approximately 30 to 40 mg were incubated in 1 ml Newman and Tyell serum-free media (Gibco-BRL) with 40 μCi ^35^SO_4_^2- ^for 24 hours at 37°C. At the end of this incubation, the media was recovered and stored at -20°C. The cartilage fragments were subsequently incubated in 1 ml 0.5 M NaOH at 4°C with gentle agitation for 24 hours to extract the proteoglycans. At the end of this incubation, the media was recovered and stored at -20°C. Unincorporated ^35^SO_4_^2- ^from media was chromatographically separated using PD-10 columns (Pharmacia, Piscataway, NJ, USA), and radiolabeled GAGs released into the culture media or recovered from the alkaline treatment were quantified by scintillation counting, as described previously [39].

### Histology

On day 7, the rabbits were euthanized by Beuthanasia-D (Schering Plough Animal Health Corp., Union, NJ, USA) overdose, and the knee joints collected. Synovial capsules were immersion-fixed in 10% buffered formalin for several days. The fixed tissues were then dehydrated in a gradient of alcohols, paraffin embedded, sectioned at 5 μm, mounted on glass slides, and then stained using hematoxylin and eosin. Sections were examined by light microscopy at 20× magnification.

### Statistical analysis

Data were analyzed using the Microsoft Excel graphing and statistical program. The Student's *t *test assuming unequal variances were performed to determine significant differences between groups. *P *< 0.05 was considered statistically significant.

## Results

### Expression of hIL-10, vIL-10, and mut.hIL-10 after intra-articular injection of recombinant adenovirus into the rabbit knee

**Figure 1:**
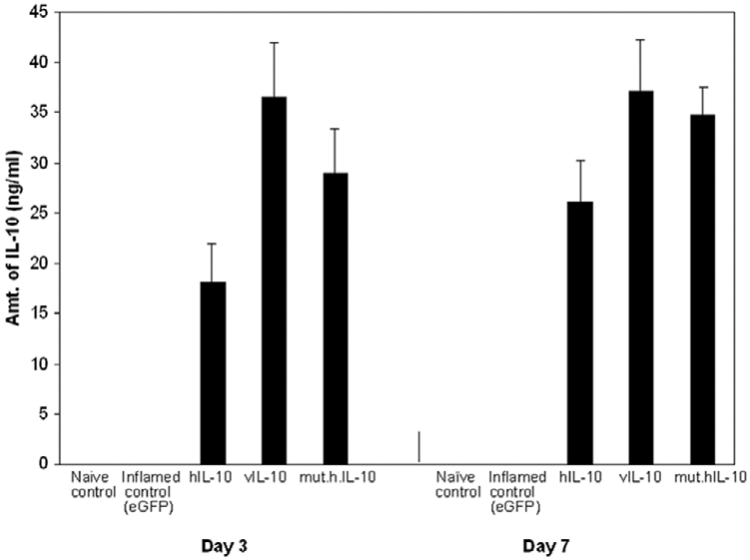
Intra-articular expression of human IL-10 (hIL-10), viral IL-10 (vIL-10), and mutant human IL-10 (mut.hIL-10). Twenty-four hours post antigen-induced arthritis (AIA) induction, 5 × 10^9 ^particles of Ad.hIL-10, Ad.vIL-10, Ad.mut.hIL-10 or adenovirus expressing enhanced green fluorescent protein (Ad.eGFP; as control) were injected into knees. At days 3 and 7 after injection of the virus, the knees were lavaged and levels of hIL-10, vIL-10, and mut.hIL-10 expression determined in the recovered lavage fluids by a cytokine ELISA kit. All values shown represent the mean ± standard error of the mean.

To compare the therapeutic effects of hIL-10, vIL-10, and mut.hIL-10 in a rabbit knee model of antigen induced arthritis, disease was induced by intra-articular injection of OVA into the knees of OVA immunized rabbits. Twenty-four hours post induction, 5 × 10^9 ^particles of first generation Ad.5-based vectors encoding either hIL-10, vIL-10, mut.hIL-10 or eGFP under the regulation of the cytomegalovirus enhancer/promoter were injected intra-articularly into rabbit knees. A naïve control group of rabbits was also included. Lavages were performed using saline on days 3 and 7 after adenoviral delivery, and the levels of hIL-10, vIL-10, and mut.hIL-10 in the recovered lavage fluids determined using a cytokine ELISA. ELISA measurements showed similar levels of expression of hIL-10, vIL-10, and mut.hIL-10 (Figure 1). IL-10 was not detected in sera (data not shown) of any of the treated animals or in the lavage fluids of knees that received Ad.eGFP.

### Effect of hIL-10, vIL-10, and mut.hIL-10 expression on joint inflammation

**Figure 2:**
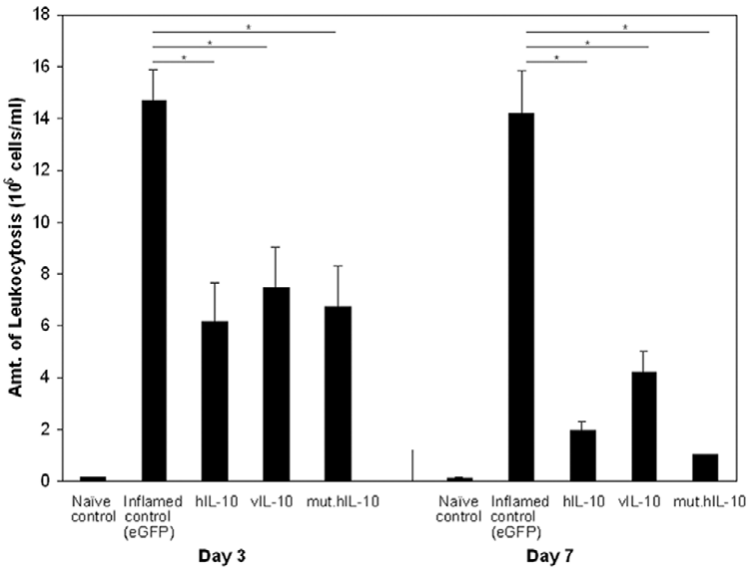
Effect of human IL-10 (hIL-10), viral IL-10 (vIL-10), and mutant human IL-10 (mut.hIL-10) on leukocytic infiltration. Twenty-four hours after antigen-induced arthritis (AIA) initiation, rabbits received Ad.hIL-10, Ad.vIL-10, Ad.mut.hIL-10 or adenovirus expressing enhanced green fluorescent protein (Ad.eGFP). On days 3 and 7 after adenovirus delivery, the knees were lavaged and the number of leukocytes counted in the recovered lavage fluid using a hemocytometer. Naïve rabbit control leukocyte levels averaged about 10^4 ^cells/ml. All values are represented as the mean ± standard error of the mean. Asterisks denote values that differ at *p *< 0.05 (Student's *t *test).

Leukocytosis is one quantitative measure of inflammation. To test and compare the ability of hIL-10, vIL-10, and mut.hIL-10 to inhibit inflammation in the inflamed rabbit knees, the number of leukocytes in the lavage fluids at days 3 and 7 were determined. The arthritic knee joints that received Ad.eGFP exhibited severe joint inflammation with a mean level of infiltrating leukocytes exceeding 15.16 × 10^6 ^cells per ml of lavage fluid at day 3, and exceeding 14.86 × 10^6 ^cells per ml of lavage fluid at day 7 (Figure 2). In comparison, lavage fluid from joints that received Ad.hIL-10 showed an average of 6.14 × 10^6 ^leukocytic cells per ml, a 60% reduction on day 3 with the number of cells reducing to 1.92 × 10^6 ^per ml, an 87% reduction by day 7. Similarly, leukocytic infiltrates from Ad.vIL-10 and Ad.mut.hIL-10 knees showed 51% and 55% reduction on day 3, and 72% and 93% reduction by day 7, respectively. The control, naïve rabbit knees showed an average of 10^4 ^leukocytes per ml.

### Effect of hIL-10, vIL-10, and mut.hIL-10 expression on cartilage matrix metabolism

**Figure 3:**
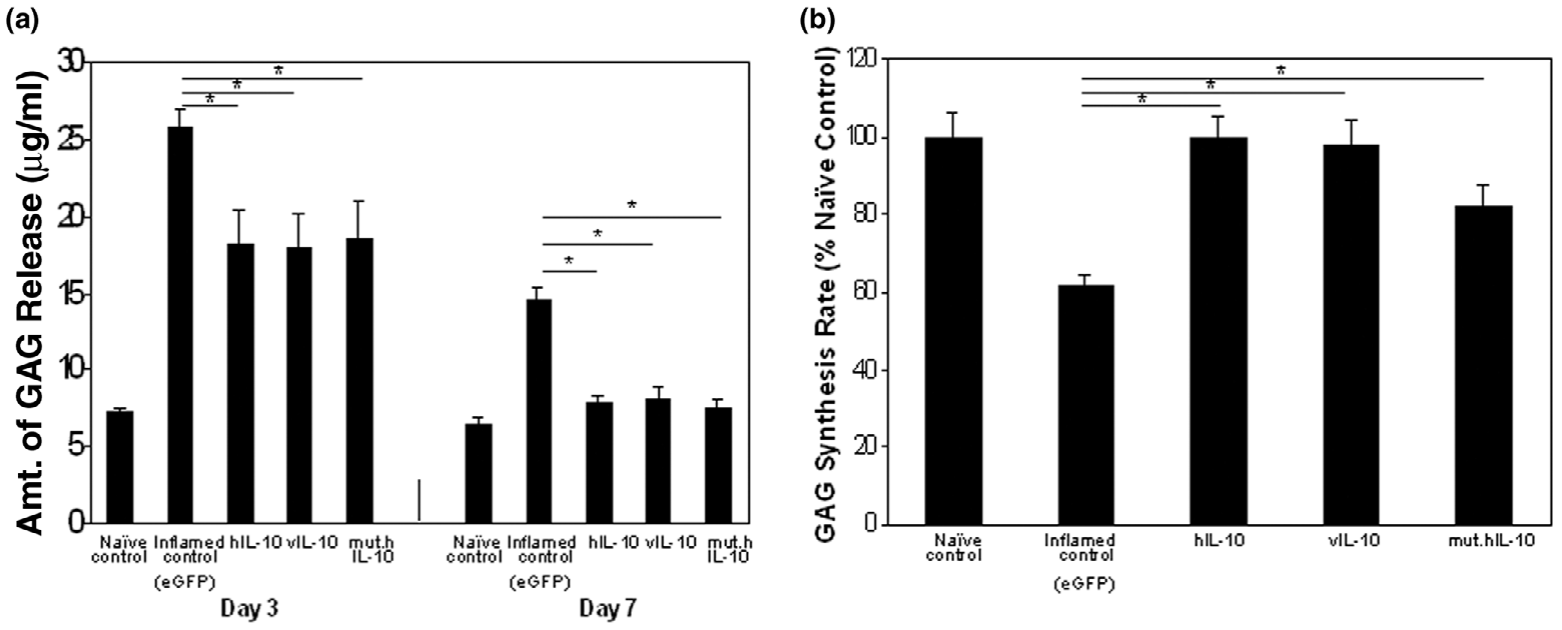
Effect of human IL-10 (hIL-10), viral IL-10 (vIL-10), and mutant human IL-10 (mut.hIL-10) on cartilage matrix metabolism. Twenty-four hours after antigen-induced arthritis (AIA) induction, rabbit knees received either Ad.hIL-10, Ad.vIL-10, Ad.mut.hIL-10 or adenovirus expressing enhanced green fluorescent protein (Ad.eGFP). At days 3 and 7, the knees were lavaged, and on day 7 the animals sacrificed and their knee joints collected. **(a) **As a measure of proteoglycan breakdown, the glycosaminoglycans (GAGs) released into the lavage fluids, were measured spectrophotometrically. **(b) **To measure the rates of proteoglycan synthesis, pieces of articular cartilage from the femoral condyles were used, and their *in vitro *^35^S0_4_^2- ^incorporation into macromolecular material measured. All values are expressed as the mean ± standard error of the mean. Asterisks denote values that differ at *p* < 0.05 (Student's *t *test).

GAG release into the synovial fluid is used as an index to determine cartilage matrix degradation. As shown in Figure 3a, hIL-10, vIL-10, and mut.hIL-10 repressed proteoglycan breakdown equally. The arthritic control knees receiving Ad.eGFP had very high levels of GAGs released into their lavage fluids whereas, on day 3, the Ad.hIL-10, vIL-10 or mut.hIL-10 treated knees showed a significant reduction of approximately 30%. By day 7, this reduction had further increased to approximately 46%, a level that was similar to naïve rabbit knees.

GAG synthesis is another parameter of cartilage matrix metabolism. To determine and compare the ability of hIL-10, vIL-10, and mut.hIL-10 to synthesize GAGs, ^35^S incorporation into proteoglycans of articular cartilage from femoral condyles was measured at day 7. GAG synthesis in arthritic, Ad.eGFP treated control knees was 61% of naïve joints (Figure 3b). In contrast, the level of GAG synthesis in knees that received Ad.hIL-10 was 99.5%, Ad.vIL-10 was 97%, and Ad.mut.hIL-10 was 82% that of naïve control knees.

### Histological analysis

**Figure 4:**
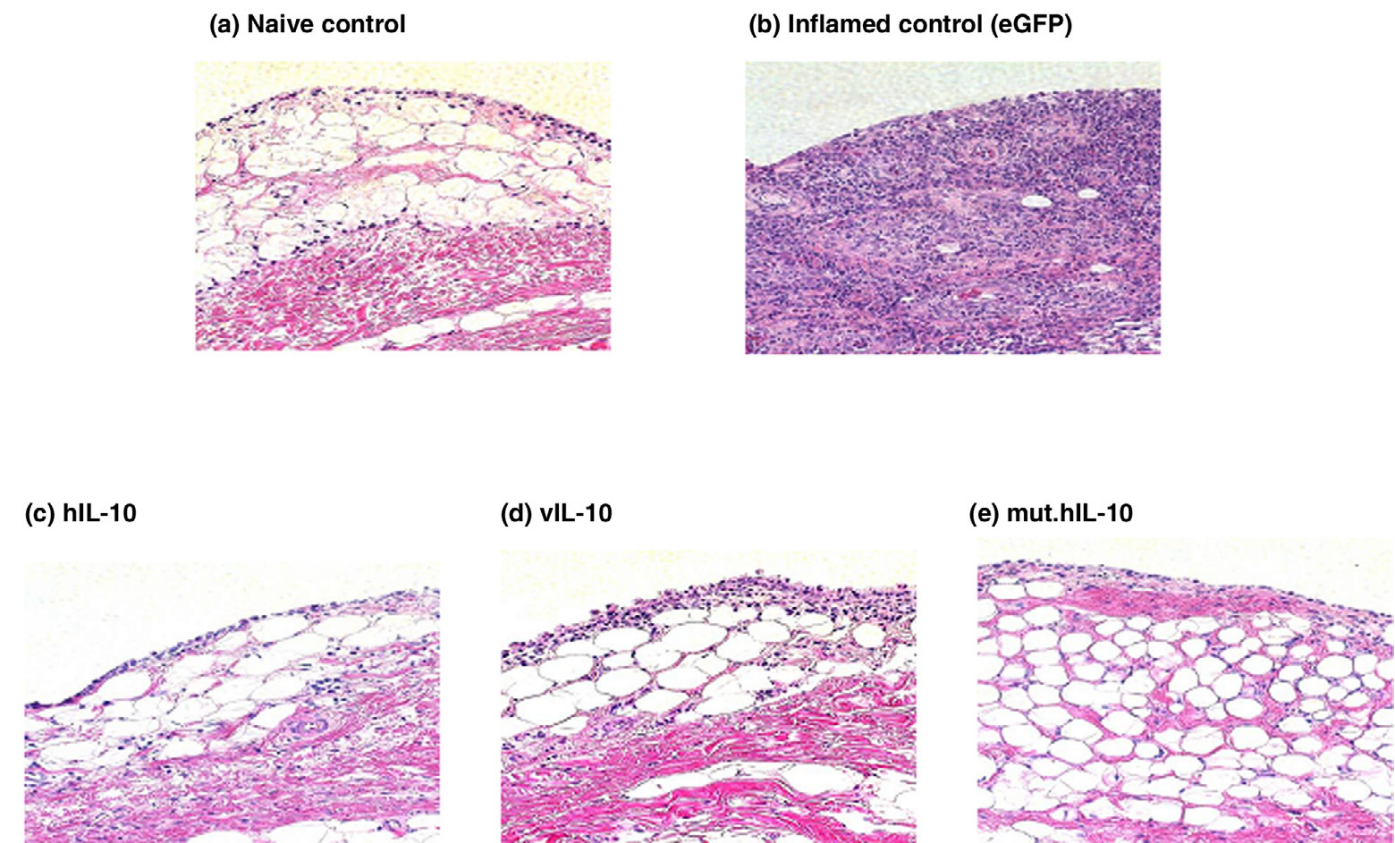
Histological analysis of synovial tissue recovered from the rabbit knees. Twenty-four hours after antigen-induced arthritis (AIA) induction, rabbits received either Ad.hIL-10, Ad.vIL-10, Ad.mut.hIL-10 or Ad.eGFP. On day 7 after the adenoviral delivery, the rabbits were sacrificed, and synovial tissue harvested, fixed, sectioned, and stained with hematoxylin and eosin. **(a) **Synovium from a naïve control rabbit knee. **(b) **Synovium from an AIA rabbit knee that received Ad.eGFP as an inflamed control. **(c) **Synovium from an AIA rabbit knee that was treated with Ad.hIL-10. **(d) **Synovium from an AIA rabbit knee that was treated with Ad.vIL-10. **(e) **Synovium from an AIA rabbit knee that was treated with Ad.mut.hIL-10. hIL-10, human IL-10; mut.hIL-10, mutant human IL-10; vIL-10, viral IL-10; eGFP, enhanced green fluorescent protein.

Histological analysis was done on tissues obtained from naïve and AIA rabbit knee joints (Figure 4). Compared to the naïve rabbit knee tissue (Figure 4a), sections from arthritic control knees that received Ad.eGFP appeared to have acute synovitis, typical of AIA (Figure 4b). The synovium was highly thickened, fibrous, hypertrophic, and hyperplasic due to excessive proliferation of synovial cells and infiltration by mononuclear leukocytes. Tissues obtained from AIA rabbit knees treated with Ad.hIL-10, Ad.vIL-10 or mut.hIL-10 (Figure 4c–e) were all more or less identical to the naïve control tissue, suggesting that Ad.hIL-10, Ad.vIL-10, and Ad.mut.hIL-10 were fairly efficacious in halting the progression of disease.

## Discussion

IL-10 is an important multifunctional cytokine that mediates the inflammatory response. The human homologue is immunostimulatory, immunosuppressive or anti-inflammatory depending upon the target tissue [15], whereas the viral homologue appears to be predominantly immunosuppressive and anti-inflammatory [27]. The biologically active mut.hIL-10 with a substituted alanine at position 87 has only immunosuppressive and anti-inflammatory capabilities similar to vIL-10 [28]. Experiments have shown that mut.hIL-10, like vIL-10, prevents mast cell proliferation and prolongs allograft survival in mice [28,30].

Previous studies have examined the effects of vIL-10 in the AIA rabbit model as well as the collagen induced arthritis mouse model showing efficacious results for treatment [11,12,32,33]. However, the cellular form of IL-10 has not been examined in the AIA model, and vIL-10 and mut.hIL-10 not compared in either rabbit or rodent models. In addition, it has been speculated that since mut.hIL-10 only has immunosuppressive and anti-inflammatory characteristics, it might be a superior cytokine for therapy of inflammatory diseases, as it would be less antigenic than vIL-10.

In this report, we have examined and compared the therapeutic potential of hIL-10, vIL-10, and mut.hIL10 in the AIA rabbit model. We have demonstrated that local adenovirus-mediated intra-articular gene transfer of hIL-10, vIL-10 or mut.hIL-10 in the rabbit knees resulted in significant improvements in several pathologies associated with the disease. A considerable decrease in the number of intra-articular infiltrating leukocytes as well as the degree of synovitis in the knee joints was observed. In addition, these cytokines displayed not only a chondroprotective effect in blocking cartilage matrix breakdown but also a chondrogenic effect in maintaining new cartilage matrix synthesis. Interestingly, our data suggest that hIL-10 effectively blocked the progression of the disease in the knees of the AIA rabbits despite its immunostimulatory function. Furthermore, the mutant form of hIL-10 was as effective as vIL-10 in inhibiting the AIA in rabbits, but not more effective than wild-type IL-10.

A previous study from our group has shown that TNF-α levels in rabbit arthritic knees were reduced by vIL-10 treatment [11]. Additional studies have shown vIL-10 to be able to inhibit the production of pro-inflammatory cytokines such as TNF-α and T cell growth factors, as well as block antigen presentation on macrophages and dendritic cells [20-24]. Thus, it is likely that the therapeutic effects of hIL-10 and mut.hIL-10 are also due partially to the suppression of TNF-α, or blockage of antigen presentation on antigen-presenting cells.

The application of gene therapy represents a novel approach for the treatment of RA, overcoming obstacles of protein delivery while providing a sustainable high efficacy and greater safety. Multiple studies in different animal models with direct viral gene transfer of therapeutic agents provide proof supporting the use of gene therapy in arthritis [9-13,32,33], and a recent phase I clinical trial using IL-1Ra by *ex vivo *gene transfer to arthritic joints was also successfully completed [40,41]. Systemic non-viral delivery methods, such as administration of engineered syngeneic fibroblasts [42], and intranasal delivery of plasmid DNA [43] also show promise.

Although shown in several experimental animal models to be an effective therapeutic for arthritis [11,12,32,33], vIL-10 has not been considered for clinical gene therapy use. It is a foreign protein and would consequently generate a neutralizing immune response undermining its potential as a therapeutic. However, hIL-10 and presumably mut.hIL-10 would be recognized as self and would escape the immune response. Both these cytokines have shown significant therapeutic efficacy in our study using the AIA rabbit model and could hence have considerable potential for development of clinical gene therapy approaches for RA.

## Conclusion

In this report, we demonstrate by adenoviral-mediated intra-articular gene transfer to the rabbit knee that hIL-10, vIL-10, and mut.hIL-10 are all similarly effective in blocking the progression of antigen induced arthritis. In particular, the three forms of IL-10 were all successful in reducing intra-articular leukocytosis and the degree of synovitis, as well as normalizing cartilage matrix metabolism. These results demonstrate that hIL-10 and mut.hIL-10, which are non-immunogenic compared to vIL-10, would be as efficacious as vIL-10 in treating arthritis pathologies following intra-articular gene transfer.

## Abbreviations

Ad. = adenovirus; AIA = antigen-induced arthritis; ELISA = enzyme-linked immunosorbent assay; GAG = glycosaminoglycan; hIL-10 = human IL-10; IL = interleukin; IL-1Ra = interleukin-1 receptor antagonist; mut.hIL-10 = mutant human IL-10; OVA = ovalbumin; RA = rheumatoid arthritis; TNF = tumor necrosis factor; vIL-10 = viral IL-10.

## Competing interests

The University of Pittsburgh has patented gene therapy approaches for treating arthritis. PDR is a Scientific Advisory Board member of a company that has licensed the technology.

## Authors' contributions

AK and ERL performed the construction of the adenoviral vectors and analysis in the rabbit model of AIA and AK wrote the initial draft of the manuscript. JN and ZM assisted in the therapeutic analysis in the rabbit model. PDR conceived of the study and participated in its design and helped to edit the manuscript.
